# Landscape of Invasive Fusariosis in Pediatric Cancer Patients: Results of a Multicenter Observational Study From Latin America

**DOI:** 10.1093/ofid/ofae285

**Published:** 2024-05-17

**Authors:** Fabianne Carlesse, Adriana Maria Paixão de Sousa da Silva, Jaques Sztajnbok, Nadia Litivinov, Karina Peron, Marcelo Otsuka, Mariana Volpe Arnoni, Marcelo Schirmer, Patricia de Oliveira Costa, Ana Lucia Munhoz Cavalcanti de Albuquerque, Hugo Morales, Eduardo Lopez-Medina, Carlos A. Portilla, Romina Valenzuela, Fabrizio Motta, Fabio Araújo Motta, João Nobrega de Almeida Junior, Maria Elena Santolaya, Arnaldo Lopes Colombo

**Affiliations:** Instituto de Oncologia Pediátrica—IOP-GRAACC-UNIFESP, Departamento de Pediatria, São Paulo, Brazil; Departamento de Pediatria, Universidade Federal de São Paulo, São Paulo, Brazil; Instituto de Oncologia Pediátrica—IOP-GRAACC-UNIFESP, Departamento de Pediatria, São Paulo, Brazil; Instituto de Tratamento do Cancer Infantil (ITACI), Instituto da Criança do Hospital das Clínicas da Faculdade de Medicina da Universidade de São Paulo, São Paulo, Brazil; Instituto de Infectologia Emilio Ribas, Intensive Care Unit, Department of Emergency Medical Care, São Paulo, Brazil; Instituto de Tratamento do Cancer Infantil (ITACI), Instituto da Criança do Hospital das Clínicas da Faculdade de Medicina da Universidade de São Paulo, São Paulo, Brazil; Instituto de Tratamento do Cancer Infantil (ITACI), Instituto da Criança do Hospital das Clínicas da Faculdade de Medicina da Universidade de São Paulo, São Paulo, Brazil; Hospital Infantil Darcy Vargas, São Paulo, Brazil; Hospital Infantil Darcy Vargas, São Paulo, Brazil; Instituto Nacional do Cancer—INCA, Department of Pediatrics, Rio de Janeiro, Brazil; Instituto Nacional do Cancer—INCA, Department of Pediatrics, Rio de Janeiro, Brazil; Hospital Federal da Lagoa Rio de Janeiro, Department of Pediatrics, Rio de Janeiro, Brazil; Hospital Erasto Gaertner, Department of Pediatrics, Curitiba, Brazil; Centro de Estudios en Infectología Pediátrica CEIP, Department of Pediatrics, Universidad del Valle, Clínica Imbanaco, Grupo Quironsalud, Cali, Colombia; Centro de Estudios en Infectología Pediátrica CEIP, Department of Pediatrics, Universidad del Valle, Clínica Imbanaco, Grupo Quironsalud, Cali, Colombia; Faculty of Medicine, Hospital Dr Luis Calvo Mackenna, Universidad de Chile, Santiago, Chile; Santa Casa de Misericórdia de Porto Alegre, Department of Pediatrics, Porto Alegre, Brazil; Hospital Pequeno Príncipe, Curitiba, Brazil; Departamento de Medicina- Escola Paulista de Medicina, Universidade Federal de São Paulo-UNIFESP, São Paulo, Brazil; Antimicrobial Resistance Institute of São Paulo (ARIES), Departamento de Medicina, UNIFESP, São Paulo, Brazil; Faculty of Medicine, Hospital Dr Luis Calvo Mackenna, Universidad de Chile, Santiago, Chile; Departamento de Medicina- Escola Paulista de Medicina, Universidade Federal de São Paulo-UNIFESP, São Paulo, Brazil; Antimicrobial Resistance Institute of São Paulo (ARIES), Departamento de Medicina, UNIFESP, São Paulo, Brazil

**Keywords:** febrile neutropenia, fusariosis, *Fusarium*, opportunistic fungal infections, pediatric cancer

## Abstract

Invasive fusariosis (IF) is a life-threatening opportunistic infection that affects vulnerable hosts. We conducted a multicenter and multinational retrospective study to characterize the natural history and clinical management of IF in pediatric cancer patients. We selected patients <18 years old who were sequentially hospitalized in 10 Latin American medical centers with a diagnosis of IF between 2002 and 2021. Data were collected using an electronic case report form complemented by a dictionary of terms. We assessed mortality rates at 30, 60, and 90 days. We collected data from 60 episodes of IF (median age, 9.8 years) that were mostly documented in patients with hematologic cancer (70%). Other risk conditions found were lymphopenia (80%), neutropenia (76.7%), and corticosteroid exposure (63.3%). IF was disseminated in 55.6% of patients. Skin lesions was present in 58.3% of our patients, followed by pulmonary involvement in 55%, sinusitis in 21.7%, bone/joint involvement in 6.7% and 1 case each of endocarditis and brain abscess. Positive blood and skin biopsy cultures were detected in 60% and 48.3% of cases, respectively. *Fusarium solani* complex was the most commonly identified agent (66.6%). The majority of patients received monotherapy within the first 72 hours (71.6%), either with voriconazole or amphotericin B formulation. The mortality rates at 30, 60, and 90 days were 35%, 41.6%, and 45%, respectively. An important factor affecting mortality rates appears to be disseminated disease. The high percentage of patients with fungal involvement in multiple organs and systems highlights the need for extensive workup for additional sites of infection in severely immunocompromised children.

## Teaser

Invasive fusariosis is a devastating disease in children, for which an intensive diagnostic workup is mandatory to check for fungal deep-seated infection in different organs. Mortality rates remain high despite the frequent use of combined antifungal therapy.

*Fusarium* spp are ubiquitous saprophytic molds capable of infecting humans, leading to a wide range of clinical manifestations, depending on the host's immune status, portal of entry, and virulence attributes of the pathogen [1]. Despite the remarkable genetic diversity exhibited by *Fusarium* spp, approximately 80% of all human infections are caused by members of the *Fusarium solani* and *Fusarium oxysporum* species complexes [2, 3].

In vulnerable hosts, especially neutropenic patients with acute leukemia and patients with severe T-cell immunodeficiency, invasive fusariosis (IF) is one of the leading mold infections causing multiple organ involvement and high mortality. In this regard, IF has been also documented in nonneutropenic hematopoietic stem cell transplant recipients, mainly those who develop graft-vs-host disease, requiring T-cell immunosuppression [4]. Indeed, corticosteroid therapy significantly affects fusariosis outcomes, with a notably higher death rate reported among corticosteroid-treated patients compared with nonrecipients [5].

The outcome of IF is strongly affected by the severity of the host’s underlying condition of the host and not only by the prompt initiation of antifungal therapy. In this scenario, the immune reconstitution of the patient is a mandatory step to increase survival [1, 4, 5].

Scarce data are available on the natural history of IF in pediatric patients, as well as from clinical trials of different therapeutic strategies against *Fusarium* spp. in this particular population [6–8]. Consequently, we continue to extrapolate principles on the clinical management of IF from adult to pediatric patients without clear evidence of benefits. The purpose of the current study was to characterize the main clinical and epidemiologic characteristics of IF in a multicenter and multinational protocol conducted in 10 medical centers from 3 Latin American countries.

## METHODS

### Study Design

This was a cross-sectional multicenter and multinational retrospective cohort study enrolling all pediatric cases of proven IF (in patients <18 years old) that were sequentially documented in 10 medical centers from Brazil, Chile, and Colombia between 2002 and 2021. The screening of putative cases of IF by local investigators was based on a systematic review of medical discharge summaries and laboratory reports, with emphasis on blood and tissue biopsy cultures obtained throughout the period of study. Only cases of proven IF were included, following the European Organization for Research and Treatment of Cancer/Mycoses Study Group criteria [9]. Some cases had already been published as small series or outbreak reports, and they were incorporated into this study with additional details to strengthen the robustness of the data [10–12].

The investigators collected data by using a standardized electronic case report form (complemented by a dictionary of terms) containing pertinent epidemiologic (demographics, underlying conditions, comorbid conditions, drug expositions), clinical (signs and symptoms, clinical management), and laboratory information (cultures, biomarkers, imaging). Electronic case reports were reviewed by 2 investigators (F. C. and A. L. C.) to check for accuracy and completeness. The first 90 days after diagnosis were reviewed. We report mortality rates at 30, 60, and 90 days after IF diagnosis.

Cultures were identified at the genus level by using conventional micromorphology. Species complex identification was achieved by additional methods, such as matrix-assisted laser desorption/ionization–time of flight mass spectrometry and/or translation elongation factor 1-α amplicon sequencing analysis when available [13]. The protocol was approved by the ethical review committees of all participating centers, with waiver of informed consent due to the observational and retrospective character of the study.

### Definitions

The time of diagnosis was considered the day when a biological sample providing the first culture positive for *Fusarium* spp was collected. Sites of infections were defined based on site-specific cultures or histopathology. Pulmonary lesions were accepted as IF manifestations on the basis of imaging findings and absence of other causes in patients with other sites positive for *Fusarium* (eg, positive blood or skin culture). Disseminated fusariosis was defined as the involvement of ≥2 noncontiguous sites as well the presence of multiple skin lesions in different sites of the body. Fungemia caused by *Fusarium* spp was not defined as disseminated disease unless another organ was involved [6].

Neutropenia was defined as an absolute neutrophil count <500/μL and lymphopenia as a lymphocyte count <1000/μL at the time of IF diagnosis [14, 15]. Corticosteroid use was considered significant if 2-mg/kg prednisolone or the equivalent was administered for ≥14 days during the 60 days before IF diagnosis. First-line antifungal therapy was defined as the drug prescribed within 72 hours after IF diagnosis. Treatment of episodes with ≥2 antifungals concomitantly for ≥72 hours was considered combined therapy [16]. Early combined therapy was defined as the initiation of ≥2 or more antifungals with activity against *Fusarium* spp. within the first 72 hours after IF diagnosis.

### Statistical Analysis

Analyses were performed with SPSS software (version 24; IMB). Categorical variables were expressed as percentages, and continuous variables as median with interquartile range (IQR). Differences between groups were evaluated using χ^2^, Fisher exact, or the Mann-Whitney *U* tests. For analyses of prognostic factors, only variables associated with a higher 60-day mortality risk (*P* < .1) on a univariate basis were introduced into the multivariate model, and backward elimination at a significance level of 5% was used for the final analysis. Kaplan-Meier statistics were used for survival analysis. Differences were considered statistically significant at *P* < .05 (2 tailed).

## RESULTS

### Patient Characteristics and Risk Conditions Related to IF

A total of 60 children with 60 episodes of IF were included in the study. Most were from Brazil (n = 54), where 8 medical centers actively enrolled patients, followed by Chile (n = 3) and Colombia (n = 3). Thirty patients (50%) were male, and the median age (IQR) of the whole cohort was 9.8 (5.7–14–6) years (Table 1). Children <3 years of age accounted for 13.3% of total patients (n = 8).

**Table 1.** Demographic and Clinical Characteristics of 60 Children With Invasive Fusariosis Documented in 10 Latin American Medical Centers (2002–2021)

Characteristics	Patients, No. (%)^a^ (N = 60)
Demographics	
Age, median (IQR)	9.8 (5.7–14–6)
Male sex	30 (50)
Underlying disease	
Hematologic cancer	42 (70)
Acute lymphocytic leukemia	29 (48.3)
Acute myeloid leukemia	10 (16.6)
Non-Hodgkin lymphoma	3 (5)
Aplastic anemia	4 (6.7)
Hemophagocytic lymphohistiocytosis	1 (1.7)
Solid tumors	12 (20)
Neuroblastoma	3 (5)
CNS tumors	4 (6.7)
Other solid tumors	5 (8.3)
Primary immunodeficiency	1 (1.7)
Transplant recipients	
Solid organ	1 (1.7)
Allogeneic stem cell	12 (20)
Autologous stem cell	4 (6.7)
Risk conditions	
Central venous catheter	57 (95)
Lymphopenia	48 (80)
Neutropenia	46 (76.7)
Corticosteroid exposure	38 (63.3)
Previous antifungal exposure	34 (56.7)
Clinical features	
Fever	55 (91.7)
Skin lesions	35 (58.3)
Cough	19 (31.6)
Dyspnea	13 (38.3)
Hemoptysis	4 (6.7)
Muscular pain	15 (25)
Infection site	
Skin	35 (58.3)
Lungs	33 (55)
Sinus	13 (21.7)
Bone/joint involvement	4 (6.7)
Endocarditis	1 (1.7)
CNS	1 (1.7)
Disseminated fusariosis	34 (56.6)
Concomitant infections (viral, bacterial, or fungal)	31 (51.6)

Abbreviations: CNS, central nervous system; IQR, interquartile range.

^a^Data represent no. (%) of patients unless otherwise specified.

Forty-two patients (70%) had hematologic cancer, predominantly acute lymphocytic leukemia (n = 29 [48.3%]) or acute myeloid leukemia (n = 10 [16.6%]). Twelve patients had a solid tumor (20%), with a predominance of primary central nervous system (CNS) tumors (n = 4). Hematopoietic stem cell transplantation was carried out in 16 patients (26.7%) before the diagnosis of IF, mainly allogenic transplantation (n = 12). Acute graft-vs-host disease before the diagnosis of IF was reported in 3 cases (5%) (Table 1). Most patients had neutropenia and lymphopenia at the time of IF diagnosis (n = 46 [76.7%] and n = 48 [80%], respectively) and had been exposed to corticosteroids (n = 38 [63.3%]). Of note, 57 patients (95%) had a central venous catheter (CVC) in place before IF, and 34 (56.7%) had been exposed to ≥1 antifungal before IF, mainly fluconazole (n = 19 [31.7%]) (Table 1).

### Clinical Presentation

Skin lesions were the first clinical manifestation in 52% of cases (31 of 60), followed by fever in 42% (25 of 60). Throughout the illness, fever became the main clinical manifestation and was documented in 55 patients (91.7%). The majority of cases were classified as disseminated fusariosis (n = 34 [56.6%]). Polymorphic skin lesions were noted in 35 patients (58.3%), including papules (n = 34 [56.6%]), erythematous macular lesions (n = 32 [53.3%]), and subcutaneous abscesses and ulcers (n = 31 [51.6%]). Pulmonary lesions were reported in 33 patients (55%), mainly represented by patchy alveolar infiltrates (n = 13 [21.6%]). Sinus involvement was seen in 13 patients (21.7%), and muscular pain was cited in 15 (25%). In 4 patients (6.7%), bone or joint involvement developed during IF, with lesions documented in the cervical region, femur, tibia, knee, or hip. In addition, we found 1 case each of endocarditis (1.7%) and brain abscess (1.7%). In total, 10% of all pediatric IF cases had involvement of sites other than blood, skin, and respiratory tract. Endophthalmitis was not detected in this cohort (Table 1).

### Microbiological Findings, Treatment and Outcome

Concomitant infections were reported in 31 cases (51.6%), consisting of viral infections in 8 patients (3 with respiratory infections, 3 with cytomegalovirus infections, and 1 each with polyomavirus or human herpesvirus 6), bacterial infections (17 gram-negative rods, 21 gram-positive cocci), and 9 fungal infections (Table 1). The fungal coinfections included 7 episodes of candidemia, 1 case of invasive infection by *Aspergillus fumigatus*, and 1 by Mucorales.

As illustrated in Table 2, a proven diagnosis of IF was obtained by culturing blood samples (n = 36 [60%]) and tissue biopsy samples (n = 29 [48.3%]). All patients <3 years old had a positive blood culture, while fungemia developed in 28 of 52 older patients (53.8%; *P* = .02). *Fusarium* species complex identification was performed in 27 cases (45%), showing *F solani* complex in 18 (66.6%), *F oxysporum* in 8 (29.6%), and *Fusarium verticillioides* in 1 (3.7%). Of note, serum galactomannan (GM) was detected in only 6 of 51 patients tested (11,7%) (Table 2).

**Table 2.** Microbiological Findings, Clinical Management, and Outcomes in 60 Children with Invasive Fusariosis (Latin America, 2002–2021)

Findings, Management, or Outcome	Children, No. (%)
Microbiological findings	
Positive blood cultures	36 (60)
Positive skin biopsy culture	29 (48.3)
Other positive cultures^a^	10 (16.6)
Positive histology^b^	29 (48.3)
Positive serum galactomannan	6 (11.7)
*Fusarium* spp	33 (55)
*Fusarium solani* complex	18/27 (66.6)
*Fusarium oxysporum*	8/27(29.6)
*Fusarium verticilioides*	1/27 (3.7)
Antifungal treatment	
Amphotericin B monotherapy	21 (35)
Voriconazole monotherapy	22 (36.6)
Echinocandin or fluconazole monotherapy	4 (6.7)
Early combined therapy	13 (21.6)
Combined therapy at any time	25 (41.6)
Overall mortality	
30 d	21 (35)
60 d	25 (41.6)
90 d	27 (45)

^a^Other positive cultures included lung biopsy cultures (n = 3), sinus biopsy cultures (n = 3), and others (n = 4), including tracheal aspirate, nasal wash sample, synovial aspirate, and nail scraping cultures.

^b^Positive histologic findings included skin (n = 22), bone (n = 2), and the central nervous system, sinuses, lungs, and nasal septum (each n = 1).

Most patients were treated within the first 72 hours with monotherapy consisting of amphotericin B (AMB) formulations (n = 21 [35%]) or voriconazole (n = 22 [36.6%]). The median durations of therapy for voriconazole and amphotericin B were 22 (IQR, 2–30 days) and 16 (8–37) days, respectively. Of note, at this time 4 patients did not receive effective therapy for IF (all were being treated with micafungin). All of them were further treated with voriconazole alone (n = 3) or in combination with liposomal AMB (n = 1), and no one died. Early combined therapy with amphotericin plus voriconazole was started in 13 patients (21.6%). Combined therapy at any time was used in 25 patients (41.6%) and consisted of amphotericin plus voriconazole in all patients except 1 (who received amphotericin and caspofungin). Adjunct therapy with granulocyte colony-stimulating factor and granulocyte transfusion were prescribed for 23 (38.3%) and 6 (10%) children, respectively. Ten patients (16.6%) underwent ≥1 surgical debridement procedure as part of their treatment.

The 30-day, 60-day, and 90-day overall mortality rates were 35%, 41.6%, and 45%, respectively (Table 2). Checking for historical trends in mortality, between 2002 and 2012, of 16 patients with findings analyzed, 9 (56.3%) had died by day 60 after IF diagnosis. In counterpart, 44 patients from our cohort had fusariosis diagnosed between 2012 and 2021, and 16 (36.4%) died by day 60 after diagnosis. This difference was not statistically significant (*P* = .17).

### Prognostic Factors at Day 60 of Follow up

**Figure 1.**
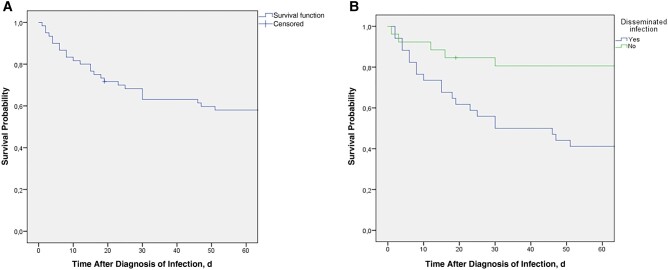
Kaplan-Meier survival curves obtained from 60 pediatric patients with invasive fusariosis (IF). *A,* Curve representing all patients with IF. *B,* Curve representing patients with disseminated fusariosis or locally invasive fusariosis.

As illustrated in Table 3, risk factors associated with the 60-day mortality rate by univariate analysis included neutropenia (*P* = .03) and disseminated disease (*P* = .002). Patients treated with voriconazole or AMB formulation monotherapy had 60-day mortality rates of 45.4% (95% confidence interval, 17.2%–73.6%) and 42.8% (14.85%–70.85%), respectively, rates that did not differ significantly from that in patients treated with the early combination of voriconazole plus an AMB formulation (46.1% [9.22%–83.08%]; *P* = .9). The 60-day mortality rate for patients who received granulocyte transfusion was 50%, not significantly different from the rate in those who did not receive this adjunctive therapy (40.7%; *P* = .7). By multivariate analysis, disseminated infection (odds ratio, 6.262) was the only independent risk factor for poor prognosis at day 60 after infection. Kaplan-Meier survival curves for the entire cohort and for the patients exposed to the single independent risk factor for mortality are shown in Figure1*A* and 1*B*.

**Table 3.** Prognostic Factors Associated With 60-Day Mortality Risk in 60 Pediatric Patients With Invasive Fusariosis

Factor	Mortality Status at 60 d, No. (%)		Univariate Analysis		Multivariate Analysis	
	Alive (n = 35)	Dead (n = 25)	*P* Value	OR	*P* Value	OR (95% CI)
Hematologic cancer	21 (60)	21 (84)	.052	3.500	…	…
Neutropenia	23 (65.7)	23 (92)	.028	6.000	…	…
Lymphopenia	26 (74.2)	22 (88)	.190	2.538	…	…
Disseminated disease	14 (40)	20 (80)	.002	6.000	.009^a^	6.262 (1585–24.744)^a^
Surgical debridement	7 (20)	3 (12)	.499	0.682	…	…
Combined therapy						
Early	7 (20)	6 (24)	.918	1.066	…	…
Any time	15 (42.8)	10 (40)	.526	0.706	…	…

Abbreviations: CI, confidence interval; OR, odds ratio.

^a^By multivariate analysis, disseminated infection was the only independent risk factor for poor prognosis at day 60 after infection.

## DISCUSSION

Literature on IF in children remains scarce and primarily limited to experiences documented as case reports or small series [11]. In the present study, we analyzed clinical, epidemiologic, and laboratory data from 60 pediatric patients with IF. Notably, mold infections seem to be more frequent in older children [11]. The median age of patients with IF was 9.8 years, with 77% >3 years old, a finding similar to the most recent publications in this setting of patients [7, 8]. As expected, the prevalence of IF was similar in both sexes.

Underlying diseases are highly determinant of the risk of patients acquiring IF. We found that 20% of cases of IF occurred in children with solid tumors, mainly CNS tumor and neuroblastoma, conditions that may require intensive chemotherapy, resulting in prolonged neutropenia and T-cell depression. This is in contrast to studies in adults, where <5% of cases occurred in patients with solid tumors [17–21].

This finding is consonant with data reported in a review of 106 cases of pediatric IF, wherein 12% of patients had a history of solid tumors [8]. Indeed, solid tumors requiring intensive chemotherapy, with long periods of immunosuppression or high doses of corticosteroids, were also found as a risk condition for 2 of 22 cases of IF published by Benish et al [7]. The results of all 3 cohorts mentioned above consolidate the concept that children with solid tumors followed by profound immunosuppression secondary to intensive chemotherapy may represent an emerging subgroup of patients susceptible to IF [7, 8].

Otherwise, similarly to adult patients, the majority of episodes of IF in our cohort were documented among children with hematologic cancer, especially acute leukemia [18, 19]. It is noteworthy that acute lymphocytic leukemia is the most documented cancer in children, especially those 2–10 years of age [22].

In adults and children, IF usually presents with fungemia, fever, skin lesions, and involvement of upper and lower respiratory tracts [7, 8, 11, 17–19]. As expected, the most common clinical findings in this cohort were fever (in 91.7% of the cases), skin lesions (58.3%), and pneumonia (55%). However, it is important to mention that in a substantial number of patients, deep-seated infections developed, affecting organs other than skin, lungs, and blood. Indeed, in 10% of our children fusariosis developed, with involvement of bones, joints, heart, and CNS. Considering the high rate of deep-seated infections reported in severely immunocompromised children with IF, as well as the fact that a substantial number of them may initially be asymptomatic [23], intensive laboratory workup is required to better support strategies of antifungal management and putative indications for surgery in this population. This high rate of deep-seated infections seems to be a peculiarity of IF in children, since osteoarticular, cardiovascular, or CNS complications of IF are apparently considered rare (occurring in <5%) in adult patients [17–19].

Consistent with the literature available for IF, diagnosis of this infection was mostly based on a positive culture obtained from blood samples (60%) and skin lesions (48.3%). For positive cultures were identified at the species level, our findings are similar to those in the literature available once *F solani* complex was the cause of most IF episodes, followed by *F oxysporum* [18, 24]. Notably, different from data generated by studies investigating the sensitivity of serum GM in adult patients with IF [25, 26], GM was detected in only 6 of 51 serum samples tested from our pediatric population. This finding needs to be further investigated in prospective studies as we may not exclude the possibility that most samples could be collected only after long antifungal therapy exposure. Indeed, we failed to find any study exploring the diagnostic value of GM specifically in pediatric patients with IF.

*Fusarium* infections are notoriously difficult to treat despite all recent medical developments and the introduction of new antifungals. According to the literature, conditions that may increase mortality rates include persistent neutropenia, pulmonary or CNS involvement, fungemia, and recent exposure to corticosteroids [4, 17, 19]. In the current study, mortality rates were 35%, 41.6%, and 45% at 30, 60, and 90 days after diagnosis, respectively. Contrasting our findings with the reported mortality rates usually documented in adults with IF, it seems that children with IF may have better outcomes [7]. Likewise, it is important to mention that 20% of our patients had a history of solid tumors, an underlying condition rarely documented in adult patients with IF. Differences in underlying conditions associated with IF may partially explain putative discrepancies in mortality rates reported in both populations.

To our knowledge, there are no randomized trials evaluating the efficacy of antifungal drugs for the treatment of IF. The most recent guideline on clinical management of infections due to rare molds, published in 2021, strongly recommends voriconazole or a lipid formu­lation of amphotericin B for the primary treatment of IF [27]. In severely immunocompromised patients with refractory IF the use of monotherapy seems insufficient, but there are no randomized controlled trials comparing outcomes obtained with monotherapy and combined antifungal therapy [12, 28, 29]. An additional challenge for the clinical management of IF in pediatric patients is represented by the difficulties in achieving adequate plasma levels of voriconazole in this population [30–32]. Taking all these arguments together, as well as the high MICs usually documented when *Fusarium* species are tested with most antifungals, a substantial number of centers use combined therapy with polyenes and voriconazole to treat pediatric patients with IF [6, 11, 12, 29, 33]. Indeed, in a review of 106 cases of pediatric fusariosis, the authors found that combined amphotericin B and voriconazole therapy was used in 27 patients (25% of all patients) [8].

In the present study, 71.6% of our patients (43 of 60) received monotherapy within 72 hours after diagnosis of the disease, mostly represented by voriconazole (22 of 60 [36.6%]). Otherwise, combined antifungal therapy at any time was used in 41.6% of the patients (25 of 60). The 60-day mortality rates were similar in patients treated with monotherapy or combined antifungal therapy (44.2% vs 40%, respectively; *P* = .7). Other authors also failed to demonstrate any positive impact of combined antifungal therapy in patients with IF [8]. However, considering that patients were not randomized before being allocated to one or other strategy of antifungal therapy, it is reasonable to consider that combined antifungal therapy was mostly selected for patients with more severe underlying conditions and critical presentation of IF.

Granulocyte transfusions have been used as an adjunct therapy with antimicrobials exhibiting variable success rates for patients developing life-threatening infections in the presence of persistent neutropenia [34]. The start time for granulocyte transfusions is apparently an important variable affecting outcome [35]. Only 4 patients (6%) in our study received such transfusions at any time, a number too small to enable any evaluation of its impact on the outcome of IF.

Despite representing the largest study of IF conducted in pediatric patients, we must highlight certain limitations stemming from the study’s retrospective nature . In this regard, some relevant information was missing at the time investigators retrospectively reviewed the clinical records of the patients enrolled. First, we could not accurately determine the duration of antifungal exposure before collection of fungal biomarkers. Second, detailed information about lung images was lacking, which precluded any analysis of radiological findings associated with IF in pediatric patients. Third, details on dose regimens of antifungal therapy with voriconazole and amphotericin B were not available for most patients. Fourth, therapeutic drug monitoring for voriconazole was performed for most patients, but the laboratory results were not available for analysis. Fifth, details of surgical interventions were not available in most reports. Sixth, we did not have access to the total number of patients with solid tumors and hematologic conditions in each center to provide prevalence rates of IF. Finally, although a minority of cases had been previously published (representing almost 30% of all cases), the present article provides better clinical and laboratory characterization of all of them.

In conclusion, our findings suggest that IF in pediatric patients is mostly documented in hematologic patients with acute leukemias, recipients of hematologic stem cell transplantation, and less frequently children with solid tumors requiring intensive chemotherapy. Besides fungemia, pneumonia, and skin lesions, bone lesions, endocarditis, and CNS involvement may develop in 10% of patients, reinforcing the need for intensive diagnostic workup to check for deep-seated infections in severely immunosuppressed children with this condition. In terms of outcomes, mortality rates remain high but seem to be lower than with adult patients, a finding that may be partially explained by differences in the underlying conditions associated with IF in both populations. As with IF in other cohorts, an important factor affecting mortality rates seems to be disseminated disease [8, 18, 36]. Despite a failure to demonstrate any advantage of combination antifungal therapy, strategies for treating children with IF should be individualized and consider the severity of illness and difficulties in achieving adequate plasma levels of voriconazole in this particular population.
